# Prognostic value of cystatin C in patients with nasopharyngeal carcinoma: a retrospective study of 1063 patients

**DOI:** 10.6061/clinics/2016(06)09

**Published:** 2016-06

**Authors:** Jing Yuan, Miao Xu, Jing Li, Ning Li, Li-Zhen Chen, Qi-Sheng Feng, Yi-Xin Zeng

**Affiliations:** I Sun Yat-sen University Cancer Center, State Key Laboratory of Oncology in South China and Collaborative Innovation Center for Cancer Medicine, Department of Experimental Research, Guangzhou, Guangdong, China; II The First Affiliated Hospital of Zhengzhou University, Department of Medical Oncology, Zhengzhou, Henan, China; III Beijing Hospital, Beijing, China

**Keywords:** Nasopharyngeal Carcinoma, Cystatin C, Survival, Prognosis

## Abstract

**OBJECTIVE:**

Patients with nasopharyngeal carcinoma experience highly variable outcomes despite receiving similar therapeutic regimens. Identifying biomarkers that predict survival and guide individualized therapy is urgently needed. Cystatin C has been explored as a valuable prognostic marker in several malignancies. We retrospectively assessed the relationship between serum cystatin C levels and nasopharyngeal carcinoma prognosis in a large cohort of nasopharyngeal carcinoma patients receiving long-term follow-up.

**METHODS:**

A total of 1063 consecutive patients diagnosed with nasopharyngeal carcinoma from June 2006 to December 2010 were retrospectively analyzed. The serum levels of cystatin C at the time of diagnosis were collected. Receiver operating characteristic curve analysis, the Kaplan-Meier method and multivariate analyses using a Cox regression model were performed to assess the correlation of cystatin C levels with overall survival, progression-free survival, distant metastasis-free survival and loco-regional recurrence-free survival.

**RESULTS:**

The median follow-up duration was 68.3 months. The optimal cut-off value of cystatin C levels for predicting death was 0.945 mg/L. Compared with the low cystatin C group, the high cystatin C group experienced significantly shorter overall survival (hazard ratio=1.47, *p*=0.050), progression-free survival (hazard ratio=1.65, *p*=0.004), distant metastasis-free survival (hazard ratio=2.37, *p*<0.001) and loco-regional recurrence-free survival (hazard ratio=2.40, *p*=0.002). Based on multivariate analysis, a high cystatin C level was identified as a significant and independent negative predictor of overall survival (hazard ratio=1.47, *p*=0.050), progression-free survival (hazard ratio=1.65, *p*=0.004), distant metastasis-free survival (hazard ratio=2.37, *p*<0.001), and loco-regional recurrence-free survival (hazard ratio=2.40, *p*=0.002).

**CONCLUSION:**

Cystatin C levels are associated with the prognosis of nasopharyngeal carcinoma patients. A high cystatin C level is an independent indicator of poor prognosis for nasopharyngeal carcinoma patients.

## INTRODUCTION

Nasopharyngeal carcinoma is a form of squamous-cell carcinoma that occurs in the upper epithelial lining of the nasopharynx 1. Epidemiological studies suggested that NPC has a remarkable geographic distribution and is especially prevalent in southern China 2. The annual incidence rate of NPC reaches approximately 30 per 100,000 in prevalent regions and this value is 50-fold higher than that in western countries 3. Although NPC is radio-sensitive, approximately one-third of NPC patients develop loco-regional recurrence and/or distant metastasis 4. Therefore, identifying novel markers for predicting the prognosis of NPC is necessary.

Cystatin C (CysC), an endogenous non-glycosylated 13 kDa inhibitor of cysteine proteases that is constitutively expressed by all nucleated cells, plays a role in the regulation of cell proliferation, differentiation and migration 5. It is encoded by the CST3 gene, which is located on chromosome 20p 6. CysC is cleared via glomerular filtration, reabsorbed and catabolized by the renal tubules, and is always useful as an ideal measure of the glomerular filtration rate (GFR). The serum CysC level is very steady and is only slightly affected by age, sex and muscle mass 7. However, the serum CysC level is elevated in patients with several types of malignances, including lung cancer 8, breast cancer 9, ovarian cancer 10, colon cancer 11, head and neck carcinoma 12, hepatoma 13 and melanoma 14. The associations between CysC levels and survival of malignancies have been explored in patients with colorectal cancer 15, Non-Hodgkin-B-cell lymphoma 16 and multiple myeloma 17. All these data indicated that CysC should be taken into consideration in cancer monitoring.

However, the potential role of CysC as a prognostic marker for NPC has not been explored. In the present study, we retrospectively assessed the relationship between serum CysC levels and NPC prognosis in a large cohort of NPC patients receiving long-term follow-up. We also performed multivariate analyses to determine whether the CysC level is an independent predictor of the survival of NPC patients.

## MATERIALS AND METHODS

### Patients

This retrospective study was performed on a cohort of 1205 consecutive patients who were newly diagnosed with stage I to IV NPC at Sun Yat-sen University Cancer Center (SYSUCC) from June 1, 2006 to December 31, 2010. This study was reviewed and approved by the Medical Ethics Committee of SYSUCC.

Patient data were retrieved from the archived patient medical records and survival data were provided by the department that performed the follow-up examinations. The collected data included age, sex, smoking status, TNM stage, histological type, treatment, time of diagnosis, time of recurrence and metastasis, and pretreatment CysC level. We adopted the seventh edition of the AJCC/UICC staging system for classification of NPC.

Potentially eligible patients had been pathologically confirmed to suffer from NPC. They also had to have received comprehensive pretreatment evaluations, including physical examinations, routine hematological and biochemical examinations, computed tomography or magnetic resonance imaging of the head and neck, chest X-ray, abdominal ultrasonography and emission computed tomography of the bone.

As the serum CysC level is regulated by renal function, we excluded those patients with abnormal renal function (GFR ≤60 ml/min/1.73 m^2^ as estimated using the Modification of Diet in Renal Disease [MDRD] formula) 18. Patients were also excluded if they had prior malignancies, previous anticancer therapy, or insufficient biochemical test results or survival data.

All patients were treated with standard curative radiotherapy with or without chemotherapy (radiation dose: 60-72 Gy for the nasopharyngeal region, 50-66 Gy for the regional lymph nodes). Most patients (79.5%) classified as stage III-IV and a minority of patients (30%) classified as stage II received a platinum-based chemotherapy regimen.

### Assessments

Serum samples were collected from all patients before treatment. The serum CysC level was measured using the Hitachi-7080 automated biochemical analyzer (Hitachi, Japan) according to the manufacturer’s instructions. The assay range of the serum CysC level is from 0.39 to 6.21 mg/L; the reference range for healthy persons is from 0.40 to 1.03 mg/L.

The following outcomes were evaluated: overall survival (OS), progression-free survival (PFS), distant metastasis-free survival (DMFS), and loco-regional recurrence-free survival (LRRFS). OS was defined as the time from diagnosis to death due to any cause. PFS was defined as the time from diagnosis to tumor progression or death due to any cause. DMFS was defined as the time from diagnosis to distant metastasis. LRRFS was defined as the time from diagnosis to loco-regional recurrence. Data for these events were censored at the last follow-up if these events did not occur.

### Statistical analysis

The qualitative variables were compared using the chi-square test or the Fisher exact test. A receiver operating characteristic (ROC) curve was used to determine the optimal cut-off value of the CysC levels resulting in the highest sensitivity and specificity. OS, PFS, DMFS and LRRFS curves were estimated using the Kaplan-Meier method. The log-rank test was used to compare survival outcomes. Hazard ratios (HRs) were calculated together with 95% confidence intervals (95% CIs). Multivariate analyses using a Cox proportional hazards model were performed to identify independent prognostic factors. Two-sided *p*-values less than 0.05 were considered to be statistically significant. All analyses were conducted using SPSS 18.0.

## RESULTS

### Baseline characteristics

**Figure 1:**
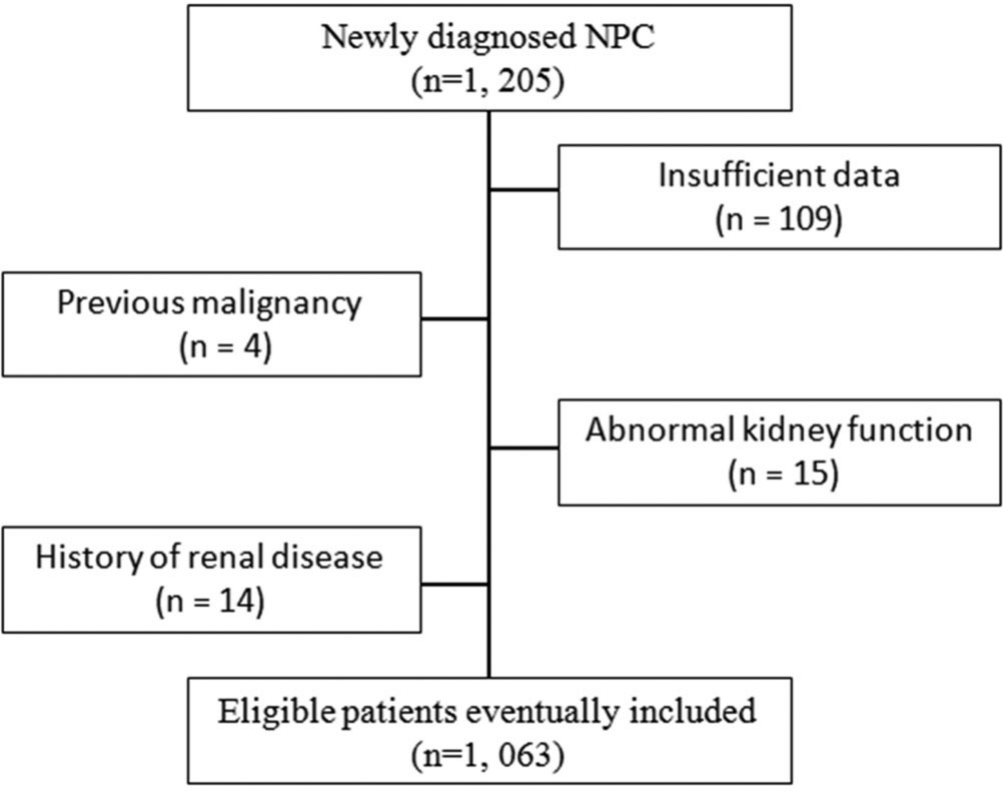
Flow diagram.

**Figure 2:**
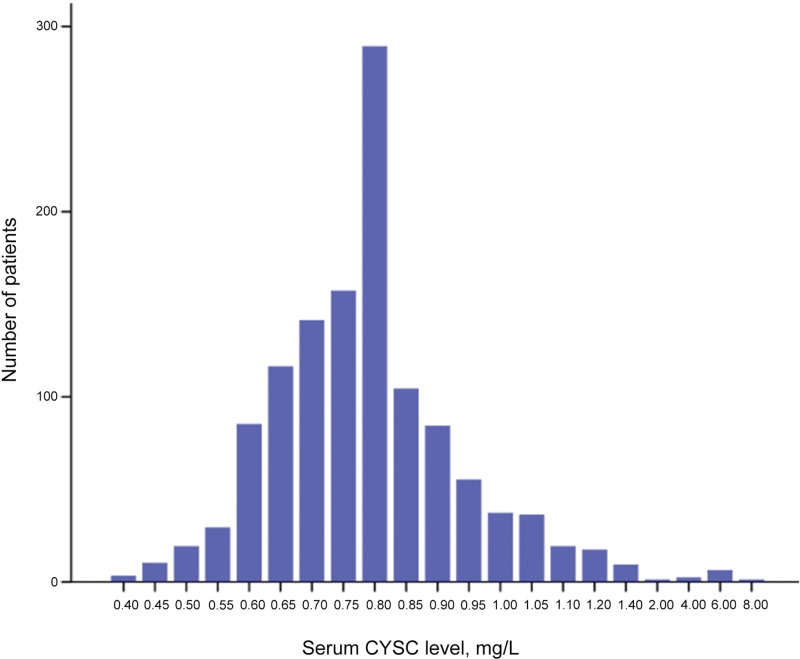
The distribution of serum cystatin C levels.

From June 1, 2006, to December 31, 2010, among 1205 potentially eligible patients, we excluded 109 patients for whom survival and hematological data were insufficient, 4 patients with another type of cancer, 15 patients exhibiting abnormal kidney function and 14 patients with a history of renal disease. A total of 1063 patients with newly diagnosed NPC classified as stage I to IV were included in our study (Figure 1). The ROC curve analyses revealed that the optimal CysC cut-off values for OS, PFS, DMFS and LRRFS were 0.945 mg/L, 0.845 mg/L, 0.925 mg/L and 0.765 mg/L, respectively. The CysC cut-off value of 0.945 mg/L was used as the uniform point for survival analyses. Thus, all patients were grouped into either the high CysC (≥0.945 mg/L) or low CysC group (<0.945 mg/L). The distribution of serum CysC levels is shown in Figure 2.

All patient characteristics are listed in Table 1. The majority of patients were male (72.0%), never-smokers (56.8%), classified as stage III-IV (80.7%), and categorized as WHO type III histological type (95.0%). The high- CysC group contained more relatively old (≥51 years) patients, more female patients and more patients who received radiotherapy alone as their primary treatment than the low CysC group. High and low CysC levels were observed in 13.3% and 86.7% of the entire cohort, respectively.

### CysC levels and survival

**Figure 3:**
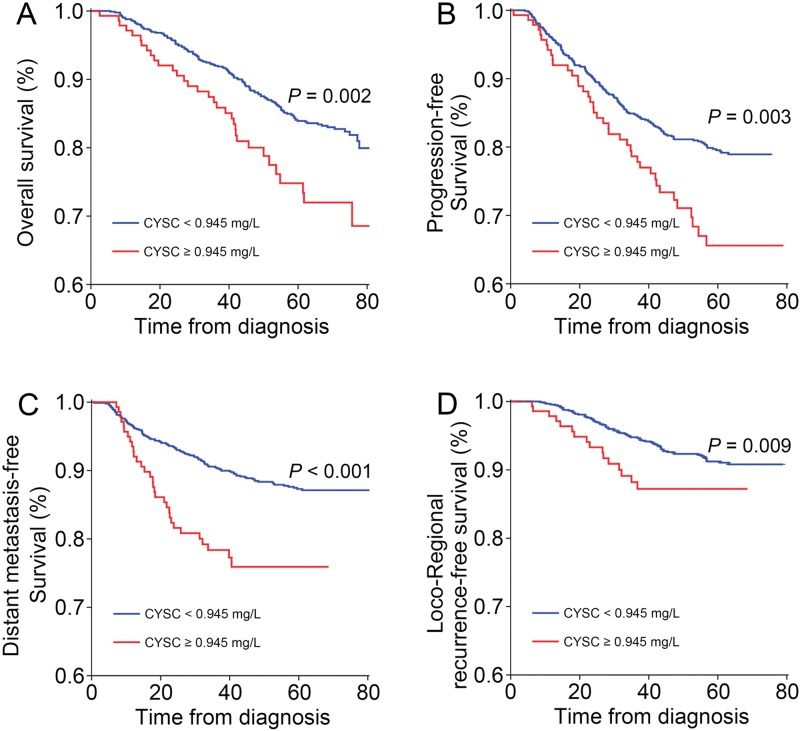
Kaplan-Meier curves for (a) overall survival, (b) progression-free survival, (c) distant metastasis-free survival, and (d) loco-regional recurrence-free survival in patients stratified according to the serum cystatin C level.

Until the end of July 2015, among the entire cohort, 218 patients experienced disease progression after primary therapy, 89 patients experienced local disease recurrence, 141 patients exhibited distant metastasis, and 176 patients had died. At the time of data cut-off, the median follow-up duration was 68.3 months (95% CI, 67.3 months to 69.4 months). OS was shorter among the patients in the high CysC group than among the patients in the low CysC group (HR 1.79, 95% CI 1.22-2.61, *p*=0.002; Figure 3A). Additionally, the high CysC group exhibited significantly shorter PFS (HR 1.67; 95% CI 1.18-2.35, *p*=0.003; Figure 3B), DMFS (HR 2.37; 95% CI 1.58-3.55, *p*<0.001; Figure 3C) and LRRFS than the low CysC group (HR 2.06; 95% CI 1.19-3.58, *p*=0.009; Figure 3D).

### Univariate and multivariate analyses assessing the prognostic value of CysC

All recorded characteristics, such as age, sex, smoking status, histological type, TNM stage, treatment modality and CysC levels, were examined via univariate analysis (Table 2). The results revealed that younger age, female gender, never smoking, early TNM stage, receiving radiotherapy alone, and a low CysC level were considered as favorable factors for OS. Moreover, female gender, early TNM stage and receiving radiotherapy alone, and a low CysC level were considered as favorable factors for PFS. Furthermore, a low CysC level was considered as a favorable factor for DMFS. Finally, older age, receiving radiotherapy alone and a low CysC level were considered as favorable factors for LRRFS. The CysC level was associated with all of the examined survival outcomes.

All factors displaying *p-*values of less than 0.1 based on univariate analyses were included in the multivariate models (Table 3). The results demonstrated that a high CysC level was a significant, independent negative predictor of OS (HR=1.47, 95% CI=1.00-2.16, *p*=0.050), PFS (HR=1.65, 95% CI=1.17-2.34, *p*=0.004), DMFS (HR=2.37, 95% CI=1.58-3.55, *p*<0.001), and LRRFS (HR=2.40, 95% CI=1.37-4.21, *p*=0.002). Additionally, age was an independent of OS and LRRFS, and female gender was an independent favorable predictor of OS and PFS. Furthermore, stage I-II was an independent favorable factor for OS and receiving radiotherapy alone was an independent favorable factor for PFS.

## DISCUSSION

In this retrospective study, which to our knowledge isthefirst to explore the prognostic value of CysC, we demonstrated that low CysC levels were associated with longer OS, PFS, DMFS and LRRFS among NPC patients. Alow CysC level was identified as a significant, favorable predictor or outcomes for patients with NPC, independent of all other examined clinico-pathological features of NPC.

There is increasing interest in the role of CysC in malignancies. The study by Kos et al. investigated the prognostic value of stefin A, stefin B and CysC in 345 patients with colorectal cancer and 125 healthy controls. They found that high CysC levels were associated with shorter survival 15. Mulaomerović et al. reported that CysC is a potential marker of disease recurrence for patients with non-Hodgkin B-cell lymphoma 16. Similarly, the study by Terpos et al. suggested that the serum CysC is an independent predictor of survival for multiple myeloma patients 17. Our results are consistent with these findings suggesting a role of CysC in cancer progression.

The mechanism underlying the association between CysC levels and NPC patient survival remains unclear. Because CysC is an inhibitor of cysteine proteases, a low CysC level facilitates invasiveness of cancer cells *in vitro* 19,20. However, in serum, a high CysC level predicts adverse outcomes. The reasons for the differences in the activities of CysC in serum and from those in tumors of cancer patients are complex. First, CysC plays a role in the regulation of cysteine proteases and other activities involved in tumor regression 21. As instinct reflexive response to cancer-induced damage, CysC is secreted into serum by immune cells. Second, cystatin family members not only act as cysteine proteases inhibitors but also function in a series of biological activities such as cell differentiation, proliferation, migration and immune regulation by stimulating nitric oxide release from macrophages and by modulating interleukin and cytokine production in T cells and fibroblasts 22-24. In the setting of malignancies, CysC may be secreted to participate in those important cancer-related biological activities in addition to inhibiting cysteine proteases.

Although the present study is certainly the first to explore whether the CysC level at the time of diagnosis is associated with NPC prognosis, several limitations of this study must be acknowledged. First, we cannot exclude other potential confounding factors such as physical activity, plasma Epstein-Barr virus DNA concentrations and family history. Second, because the prognosis of NPC is favorable, the follow-up duration for our study cohort is relatively short. Third, because our study is a single-center study of Chinese NPC patients, our results may need to be verified in other ethnic groups.

Despite these shortcomings, our findings demonstrate forthe first time that CysC levels are associated with the prognosis of NPC patients. A low CysC level is an independent factor indicating favorable prognosis for NPC patients. These findings must be validated and explored in epidemiological, biological and genetic studies.

## AUTHOR CONTRIBUTIONS

Yuan J conceived and designed the study, analyzed and interpreted the data, wrote the manuscript and conducted literature searches. Xu M conceived and designed the study and critically revised the manuscript. Li J and Li N collected and analyzed all data related to this study. Chen LZ and Feng QS reviewed the records. Zeng YX is the supervisor of the study who approved the final version of the manuscript.

## Figures and Tables

**Table 1:** Baseline clinical characteristics of the 1063 nasopharyngeal carcinoma patients according to their cystatin C levels.

	CysC level mean (SD), mg/L	CysC level		
Characteristics		<0.945 mg/L	≥0.945 mg/L	*p*-value
Age				<0.001
<51 years	0.78 (0.49)	484 (52.5)	40 (28.4)	
≥51 years	0.84 (0.40)	438 (47.5)	101 (71.6)	
Gender				0.002
Male	0.74 (0.45)	648 (70.3)	117 (83.0)	
Female	0.83 (0.44)	274 (29.7)	24 (17.0)	
Smoking status				0.138
Ever	0.84 (0.47)	390 (42.3)	69 (48.9)	
Never	0.79 (0.43)	532 (57.7)	72 (51.1)	
Histological type				0.208
WHO type I-II	0.76 (0.12)	49 (5.3)	4 (2.8)	
WHO type III	0.81 (0.46)	873 (94.7)	137 (97.2)	
Stage (7^th^ UICC/AJCC)				0.263
I-II	0.82 (0.60)	183 (19.8)	22 (15.6)	
III-IV	0.81 (0.40)	739 (80.2)	119 (84.4)	
Treatment				0.038
Radiotherapy	0.83 (0.53)	255 (27.7)	51 (36.2)	
Chemo-radiotherapy	0.80 (0.41)	667 (72.3)	90 (63.8)	

Abbreviations: CysC: cystatin C; SD: standard deviation; WHO: World Health Organization.

**Table 2:** Univariate analysis of prognostic factors for patients with nasopharyngeal carcinoma.

	OS		PFS		DMFS		LRRFS	
Factors	HR(95%CI)	*p*	HR(95%CI)	*p*	HR(95%CI)	*p*	HR(95%CI)	*p*
Gender								
Male *vs* Female	2.48(1.63-3.79)	<0.001	1.67(1.19-2.33)	0.003	1.05(0.73-1.53)	0.779	0.93(0.60-1.47)	0.77
Age (years)								
≥51 *vs* <51	1.55(1.14-2.10)	0.005	0.95(0.73-1.24)	0.715	1.19(0.86-1.66)	0.302	0.56(0.36-0.85)	0.007
Smoking status								
Ever *vs* Never	1.58(1.17-2.12)	0.003	1.27(0.98-1.66)	0.074	0.998(0.72-1.39)	0.992	1.01(0.66-1.54)	0.97
Histological type (WHO)								
Type III *vs* type I-II	0.95(0.49-1.86)	0.88	0.99(0.54-1.81)	0.97	3.76(0.93-15.20)	0.063	0.74(0.32-1.68)	0.47
Stage								
III-IV *vs* I-II	2.55(1.52-4.26)	<0.001	1.55(1.06-2.26)	0.025	0.91(0.61-1.36)	0.629	1.22(0.70-2.13)	0.48
Treatment								
CRT *vs* RT	1.64(1.14-2.35)	0.008	1.84(1.32-2.58)	<0.001	0.95(0.66-1.37)	0.785	1.72(1.01-2.92)	0.044
CysC level (mg/L)								
≥0.945 *vs* <0.945	1.79(1.22-2.61)	0.003	1.67(1.18-2.35)	0.004	2.37(1.58-3.55)	<0.001	2.06(1.19-3.58)	0.01

CI: confidence interval; OS: overall survival; PFS: progression-free survival; DMFS: distant metastasis-free survival; LRRFS: loco-regional recurrence-free survival.

**Table 3:** Multivariate analysis of independent prognostic factors for patients with nasopharyngeal carcinoma.

	OS		PFS		DMFS		LRRFS	
Factors	HR (95%CI)	*p*	HR (95%CI)	*p*	HR (95%CI)	*p*	HR (95%CI)	*p*
Gender								
Male *vs* Female	2.34 (1.53-3.58)	<0.001	1.57 (1.12-2.19)	0.009	—	—	—	—
Age (years)								
≥51 *vs* <51	1.41 (1.03-1.92)	0.031	—	—	—	—	0.51 (0.33-0.78)	0.002
Smoking status								
Ever *vs* Never	1.13 (0.83-1.56)	0.44	1.07 (0.80-1.42)	0.652	—	—	—	—
Histological type (WHO)								
Type III *vs* type I-II	—	—	—	—	3.56 (0.88-14.37)	0.075	—	—
Stage								
III-IV *vs* I-II	2.58 (1.54-4.32)	<0.001	1.21 (0.81-1.81)	0.355	—	—	—	—
Treatment								
CRT vs RT	1.38 (0.94-2.01)	0.1	1.86 (1.33-2.61)	<0.001	—	—	1.68 (0.99-2.87)	0.057
CysC level (mg/L)								
≥0.945 *vs* <0.945	1.47 (1.00-2.16)	0.05	1.65 (1.17-2.34)	0.004	2.37 (1.58-3.55)	<0.001	2.40 (1.37-4.21)	0.002

CI: confidence interval; OS: overall survival; PFS: progression-free survival; DMFS: distant metastasis-free survival; LRRFS: loco-regional recurrence-free survival.

“—” indicates that the factor was not included in the multivariate analysis because its *p*-value based on univariate analysis was greater than 0.1.
